# Citrus Psorosis Virus: Current Insights on a Still Poorly Understood Ophiovirus

**DOI:** 10.3390/microorganisms8081197

**Published:** 2020-08-06

**Authors:** Zineb Belabess, Tourya Sagouti, Naima Rhallabi, Abdessalem Tahiri, Sébastien Massart, Rachid Tahzima, Rachid Lahlali, M. Haissam Jijakli

**Affiliations:** 1 Plant Protection Laboratory. INRA, Centre Régional de la Recherche Agronomique (CRRA), Oujda 60000, Qualipole de Berkane, 63300 Berkane, Morocco; zineb.belabess@inra.ma; 2 Faculté des Sciences et Techniques de Mohammedia, Laboratoire de Virologie, Microbiologie et Qualité/Ecotoxicologie et Biodiversité, 20650 Mohammedia, Morocco; sagoutitourya@gmail.com (T.S.); rallabina@yahoo.fr (N.R.); 3 Phytopathology Unit, Department of Plant Protection, Ecole Nationale d’Agriculture de Meknès, 50001 Meknes, Morocco; atahiri@enameknes.ac.ma; 4 Integrated and Urban Plant Pathology Laboratory, Gembloux Agro BioTech, University of Liege, 25030 Gembloux, Belgium; sebastien.massart@uliege.be (S.M.); rachid.tahzima@uliege.be (R.T.); mh.jijakli@uliege.be (M.H.J.)

**Keywords:** CPsV, citrus, ophiovirus, transmission, diagnostic, PDR, RNA-silencing, VIGS, PTGS, Morocco

## Abstract

Citrus psorosis was reported for the first time in Florida in 1896 and was confirmed as a graft-transmissible disease in 1934. Citrus psorosis virus (CPsV) is the presumed causal agent of this disease. It is considered as a type species of the genus *Ophiovirus*, within the family Aspiviridae. CPsV genome is a negative single-stranded RNA (-ssRNA) with three segments. It has a coat protein (CP) of 48 kDa and its particles are non-enveloped with naked filamentous nucleocapsids existing as either circular open structures or collapsed pseudo-linear forms. Numerous rapid and sensitive immuno-enzymatic and molecular-based detection methods specific to CPsV are available. CPsV occurrence in key citrus growing regions across the world has been spurred the establishment of the earliest eradication and virus-free budwood programs. Despite these efforts, CPsV remains a common and serious challenge in several countries and causes a range of symptoms depending on the isolate, the cultivar, and the environment. CPsV can be transmitted mechanically to some herbaceous hosts and back to citrus. Although CPsV was confirmed to be seedborne, the seed transmission is not efficient. CPsV natural spread has been increasing based on both CPsV surveys detection and specific CPsV symptoms monitoring. However, trials to ensure its transmission by a soil-inhabiting fungus and one aphid species have been unsuccessful. Psorosis disease control is achieved using CPsV-free buds for new plantations, launching budwood certification and indexing programs, and establishing a quarantine system for the introduction of new varieties. The use of natural resistance to control CPsV is very challenging. Transgenic resistance to at least some CPsV isolates is now possible in at least some sweet orange varieties and constitutes a promising biotechnological alternative to control CPsV. This paper provides an overview of the most remarkable achievements in CPsV research that could improve the understanding of the disease and lead the development of better control strategies.

## 1. Introduction

Psorosis is a widespread citrus disease in the world, causing yield, growth, and longevity reduction of infected trees which may linger for a few years before finally dying out [1]. It was first observed in Florida and California at the beginning of the 1890s. However, the disease originated in the Orient and spread among many citrus-growing countries around the world by the distribution of infected citrus species and varieties [2,3]. In citrus-growing countries applying advanced techniques, the disease has been brought under control notably through rigorous indexing, quarantine, and certification program [2,4]. However, it remains a serious problem in Argentina and Uruguay [5,6]. In Western Australia, the disease has been present for many years [7] but has only occurred occasionally on some trees in most groves [1]. Psorosis disease has also been reported in the Mediterranean basin [8] and could potentially be widespread in citrus-growing areas of Asia [4]. Although CPsV was described as the first virus disease affecting citrus and was often categorized among the five unusual and economically important citrus diseases, it was, however, one of the last to be characterized. These include citrus blight, citrus variegated chlorosis, Rio Grande gummosis of citrus, and dry root rot of citrus [9]. Huanglongbing, also known as citrus greening, is considered as one of the most destructive bacterial diseases of citrus [10,11]. The first progresses on CPsV characterization were made almost 120 years after the first report on psorosis disease on citrus [12]. Given the importance of and rapid research progress in citrus virology in recent years, this review emphasizes advances related to CPsV, one of the most serious viruses associated with citrus. It comprises reviews and research articles covering broad research areas on the characterization of the virus and its symptoms, the development of reliable and rapid diagnosis methods, potential vectors and seed transmission studies, and management strategies. A brief snapshot of the present situation of CPsV in the Mediterranean region, with an emphasis on its spread in citrus-growing areas of Morocco, is included.

## 2. Taxonomy, Genome Structure, and Organization

**Figure 1:**
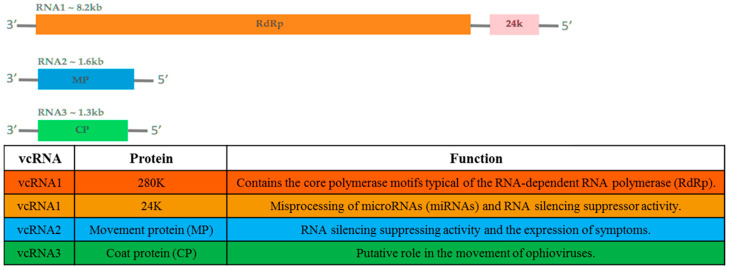
Genomic organization of citrus psorosis virus (upper panel). The three genomic RNAs are represented. Boxes indicate open reading frames (ORFs) with an indication of the encoded proteins (lower panel).

Citrus psorosis is a serious viral disease infecting citrus species in many countries [4]. The “presumed” agent of this disease, citrus psorosis virus (Aspiviridae; *Ophiovirus*; CPsV), has a tripartite (Figure 1), non-enveloped, negative-sense, single-strand RNA (-ssRNA) genome [12]. CPsV was often described as the “presumed” causal agent of psorosis since several trials for its detection by nucleic acid-based techniques and antibodies–antigen-based ones were sometimes unsuccessful and the status of infection was only based on the observation of field symptoms or reaction of indicator plants [13]. CPsV virions consist of naked filamentous nucleocapsids that are 3 nm in diameter, and 700 nm or 2000 nm long [14]. RNA1 is ~ 8184 nucleotides (nts). It contains two open reading frames (ORFs) in the viral complementary strand, separated by a 109 nts intergenic region [15]. These ORFs encode two proteins: (i) a 24kDa protein involved in the alteration in the processing of microRNAs (miRNAs)–small RNAs (sRNAs) molecules regulating gene expression in plants and animals [16] and RNA silencing suppressor activity [17], and (ii) a 280kDa protein which contains the core polymerase motifs typical of viral RNA-dependent RNA polymerases (RdRp) [15]. RNA2 is ~ 1643 nts [18,19]. It contains a single ORF in the viral complementary strand encoding a protein of ~ 54kDa [18]. This protein was shown to display several roles of viral movement protein (MP) [20] and to have RNA silencing suppressing activity as well [17]. Moreover, the MP was subsequently considered as an isolated member of the 30K superfamily with a unique structural organization [21]. It contains an aspartic protease required for autocleavage and the formation of tubule-like structures at plasmodesmata, allowing viral movement between cells [22]. The potential involvement of the 54kDa protein in symptom expression was readily highlighted [23]. RNA3, the smallest RNA, is ~1454 nts. It contains a single ORF in the viral complementary strand encoding the coat protein (CP) whose molecular weight is ~ 48kDa [18,24,25]. A potential role of CP in the movement of ophioviruses has been addressed by Robles Luna et al. [20]. The CP is localized in the cytoplasm, suggesting that virions assembly of CPsV occurs in this site [26]. The availability of the CP gene sequences, from different regions in the world, allowed performing phylogenetic analyses within CPsV isolates and with other members belonging to the genius *Ophiovirus* [27,28,29,30]. The variability of the CP gene of CPsV was assessed by analyzing 40 isolates from different geographical areas (Italy, Lebanon, Spain, and the USA) using 24 monoclonal antibodies (MABs). The serological analysis revealed 14 serogroups and at least 16 different epitopes. Furthermore, a remarkable serological variability, apparently associated with the geographical origin of the isolates, was found [31]. A second analysis conducted with 53 psorosis field sources from Italy using 23 MABs highlighted the presence of nine serogroups and at least 10 different epitopes. These serogroups were neither associated with the filed location nor with the citrus cultivar. A low genetic diversity was observed between the Italian isolates and phylogenetic analysis showed that these sources are phylogenetically distant from the CPsV isolate from Florida [27]. Analyses of 22 isolates from Argentina, California, Florida, Italy, and Spain showed the presence of two populations. The first one includes isolates from Spain, Italy, Florida, and California, whereas the second one comprises Argentinean isolates. Both groups are distant from an isolate from Texas that forms a third group alone [29]. Another study conducted by Achachi et al. [30] to examine the variability and genetic structure of the natural population of CPsV occurring in Morocco showed that genetic diversity is not structured by the geographic origin of the 34 studied isolates. Further comparisons of these isolates with samples from Spain, Italy, Argentina, California, and Florida allowed differentiation between four groups. Moroccan isolates are present in three groups (one of them includes isolate from Italy and Spain, while the two other groups contain only Moroccan isolates) and they are phylogenetically distant from the Argentinean, Floridian, Californian, and Italian isolates [30].

CPsV, the causal agent of psorosis, is the type species of the genus *Ophiovirus*, within the family Aspiviridae [12]. However, new discussions have been initiated regarding CPsV position within this genus, which contains six other species [14]. This is mainly linked to four statements: (i) CPsV is currently the only ophiovirus with a woody natural host; (ii) to date, there is no evidence of its soil transmission contrary to the obvious rule for ophioviruses known to be transmitted by the obligately parasitic soil-inhabiting fungus *Olpidium brassicae* Woronin (Olpidiaceae); (iii) no serological cross-reaction between the CP of CPsV and that of other ophioviruses has been highlighted by Western blotting; and (iv) CPsV has three and not four genome segments, as it is the case for the six other ophioviruses. All these differences once confirmed, could lead to the placement of CPsV in a different genus within the family Aspiviridae [4].

## 3. Symptoms and Economical Impact

Citrus psorosis could affect various parts of the tree including the trunk, branches, leaves, and fruits, thus causing different types of damages such as growth reduction, the appearance of thin foliage, low fruit-bearing, and tree decline. CPsV-infected trees in the orchard show typical symptoms. The most characteristic one is bark scaling in both the trunk and branches with gum production and wood discoloration below the bark lesions [12]. The major susceptible citrus varieties, which show psorosis bark scaling symptoms, are sweet orange, mandarin, and grapefruit. The sour orange, sour lemon, pomelo, and rough lemon usually do not manifest external bark symptoms. In addition to bark scaling, some foliar symptoms were reported to be associated with psorosis disease [32], including leaf flecking, vein clearing, and oak-leaf pattern [33,34,35]. It is important to emphasize that these foliar symptoms could be caused by viruses other than CPsV [3].

Although citrus psorosis progress is slow and unspectacular, it is classified as a deadly disease [36]. In light of the disease progress, CPsV-infected trees could be classified into one of four stages. Trees in stage 1 show first evidence of the disease, with the appearance of bark lesions on the trunk or one or more limbs [36]. These lesions usually begin to develop after infected trees attain an age of six years or more. Furthermore, the average age time of the first lesion development is twelve to fifteen years [37,38]. Very little dieback of the branches or deterioration of the top occurs at this stage of infection. At stage 2, one or more main limbs proving severe symptoms and bearing very few fruits appear. Regarding trees at stage 3, they are known by the loss of one-half to three-fourths of their limbs or bearing surface. These trees are considered as submarginal producers. Trees of stage 4 are usually known by the loss of most of their limbs and the production of small amounts of fruits. Since the widest range of severity of the four classes is encountered in stage 2, this stage was further divided into three sub-stages: 2A, 2B, and 2C. Trees classified as stage 2A had one main limb proving symptom. In stage 2B, trees had two or three main limbs (or up to one-third of the tree) out of production. Regarding trees in stage 2C, they are known by the destruction of up to half of their bearing surface [36]. The potential economic losses caused by CPsV to citrus producers have been assessed in a Valencia orange orchard located in California. It has been shown that the estimated reduction in yield, on trees belonging to stage 2, is 35% or by around 147 boxes of lost fruits (273 fruit boxes instead of 420 fruit boxes for normal yield from 75 infected and non-infected trees, respectively). The calculated loss of fruit in the entire orchard has been estimated after figuring the reduced yield from the remaining infected trees, blank spaces, and non-bearing replants. It was around 12% of a normal crop, which is equivalent to a complete loss of the fruit crop from 3.804 m^2^ of a 32.375 m^2^ orchard [36].

**Figure 2:**
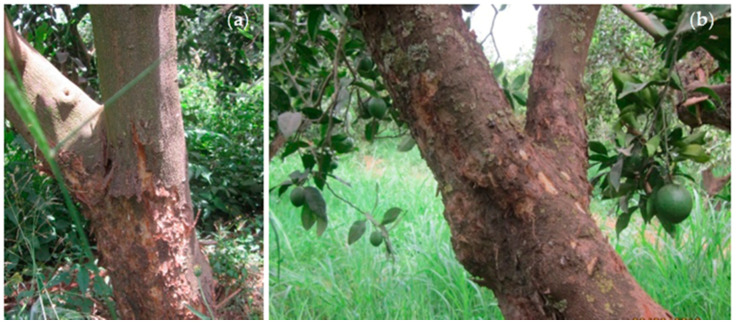
Field symptoms of citrus psorosis disease observed in Moroccan citrus orchards in the Moulouya region in 2019. (**a**) Bark scaling and gumming restricted to the stem and main branches of sweet orange trees, characteristic of psorosis A (PsA); (**b**) rampant bark scaling affecting thin branches of sweet orange trees, characteristic of PsB.

Based on the induced symptoms, two different forms of citrus psorosis disease have been later identified [32,39]. The first one is characterized by mild symptoms in which the lesions may be limited to some areas of the stem and the main branches, and is known as psorosis A (PsA). The second one is more severe and called psorosis B (PsB). The lesions induced by PsB may be rampant and affect even thin branches shedding large strips of bark (Figure 2) and cause chlorotic blotching in old leaves with the appearance of brownish gummy pustules in the leaf underside [12,23,40]. Fruits from PsB-infected trees may show depressed spots or rings in the rind with discoloration of the tissue [12]. PsB, the viral isolate associated with severe symptoms, has been referred over the years as citrus ringspot virus [41], an isolate of CPsV which is different from the Indian citrus ringspot virus (Alphaflexiviridae; *Mandarivirus;* ICRSV). CPsV distribution within the plant is not uniform [42]. The typical PsB symptoms are associated with a sequence variant of the RNA2 of CPsV. Single-strand conformation polymorphism (SSCP) analysis carried out to differentiate between PsA- and PsB-inducing CPsV sub-isolates showed identical profiles in homologous segments of RNA1 and RNA3, whereas segments of RNA2 allowed differentiation between PsA- and PsB-associated sequence variants. In other words, SSCP analysis of the RNA2 population occurring within different tissues of psorosis-infected plants showed that: (i) PsA-inducing isolates accumulate in all tissues and contain PsB-associated sequence variants at low frequency; (ii) the PsB-associated sequence variant is prevalent in blistered twigs and gummy pustules produced in old leaves, characteristic of PsB isolates; and (iii) the PsB-associated sequence variant shows a preference for accumulating in bark lesions of the trunk and limbs [23]. Overall, these results agree with those of previous studies suggesting that different CPsV isolates may be involved in citrus psorosis and ringspot diseases [43,44].

A positive correlation was observed between tree age and the number of symptomatic trees (showing bark scaling) in citrus orchards. A study carried out in three Argentina citrus groves showed that the proportion of symptomatic trees was higher in orchards that were 27 and 31 years old than in an orchard that was 17 years old [45]. However, it is important to point out that bark scaling usually appears when trees are 12–15 years old, but non-scaled psorosis-infected trees aged 20 years or more have also been found [23], especially in the absence of the severe form of the disease (PsB) [32]. Based on previous findings and conclusions reported by Velázquez et al. [23], the delayed onset of bark scaling symptoms in the trunk of trees propagated with vegetal material (buds) from PsA-infected trees (12–15 years) could be related to the low frequency of PsB-associated sequence variants in green symptomless tissues, including buds, of PsA-infected trees. Over time, and since the PsB variants prefer to accumulate in the trunk bark, their concentrations in this site raise and lead to the development of bark lesions. The late appearance of this type of symptoms was one of the factors behind the spread of CPsV in most citrus-growing areas in the world through the propagation of infected buds unknown to growers, who presumed them to be virus-free [23]. Furthermore, it has been emphasized that leaf symptoms alone and the absence or presence of bark scaling do not define psorosis [33,46,47,48,49,50]. Therefore, the presence or absence of the virus can only be confirmed by biological indexing [2,32], and antibodies–antigen- or nucleic acid-based methods [46,49].

## 4. Transmission and Epidemiology

CPsV is the first citrus viral disease showed to be transmissible by vegetative propagation and for which the first programs for eradication and budwood certification were launched to prevent its economic impacts [12,14]. In many woody species, the roots of adjacent trees graft together, forming a functional organic union through which the biological processes of one tree are highly influenced by neighboring trees by means other than simple competition. Roots are considered functionally grafted when a connection is established between them by common bark, phloem, cambium, and xylem tissues. The role of root grafting as a means of transmission of tree pathogens, including viruses, has been speculated [13]. A significant spread of two rose mosaic viruses by natural root grafting has already been reported [51]. Natural grafts of roots within a citrus orchard seem to be associated with the widespread of CPsV. This is mainly linked to the fact that citrus trees are generally planted close to each other [52]. Furthermore, statistical studies of the distribution of CPsV-infected trees in citrus orchards showed that in thirteen of fourteen examined orchards, trees adjacent to a diseased tree are more likely to be infected with the virus compared with those surrounding a healthy tree. Based on this finding, it was concluded that some natural spread of CPsV had occurred within citrus groves and that root grafting seemed to be the most likely pathway of its transmission [53].

The seedborne nature of CPsV was confirmed with the detection of viral genomic RNA in seeds of citrus infected with a CPsV isolate present in the Mediterranean region. However, CPsV was detected at significantly reduced amounts in the surface of treated seeds (dipped in hot water), suggesting that most of the virus is located externally (overall, the percentage of positive detection was 32% versus 9.5% in non-treated and treated seeds, respectively). In non-treated seeds, the virus was more prevalent in seed coats (20–83% incidence) than endosperm/embryos (3–20% incidence). The same tendency was observed in treated seeds, but positive CPsV detection was extremely low ranging from 1.4 to 35% in seed coats versus 0 to 6% in endosperm/embryos. Even though the virus was detected in the seed coats and endosperm/embryos, seed transmission was not detected from either surface-treated or untreated seeds following testing of 690 “Grosso di Puglia” mandarin and 202 “Baladi” sour orange seedlings. Therefore, although CPsV was confirmed to be seedborne, its seed transmission could have a limited influence in its spread [54].

Mechanical transmission is defined as a successful inoculation by some type of mechanical contact not involving any living vector other than man himself. It is therefore synonymous with juice, sap, hand, and manual transmission, and is different from biological, insect, graft, dodder, seed, and soil transmission [55]. Mechanical transmissions of some CPsV isolates, originating in Argentina and USA, from citrus to citrus, citrus to an herbaceous host (*Chenopodium quinoa* Willd.), or from a herbaceous host (*C. quinoa*) to another herbaceous host (*Gomphrena globosa* L.) and then back to citrus have been already reported [24,56]. The inocula has been prepared by grinding leaf tissue in a cold buffer with prechilled mortars and pestles. It has been applied thereafter immediately with cotton swabs to leaves predusted with carborundum [56]. Chlorotic to necrotic local lesions were produced in inoculated *C. quinoa* [24,56]. Some Egyptian CPsV isolates were shown as well to be mechanically transmissible from citrus to five herbaceous hosts belonging to three families (*G. globosa*, *Chenopodium amaranticolor* Coste & Reyn, *C. quinoa*, *Datura metel* L., and *Nicotiana rustica* L.). The isolates induced different symptoms and incubation periods on their host plants [57]. Mechanical transmission, as a contaminant, could be involved in some of the observed natural spread of CPsV, although experimental evidence for that was not conclusive [56].

Based on the distribution and the increasing incidence of citrus psorosis disease in the field, the hypothesis of CPsV natural dispersion, including aerial and/or soilborne vectors, has been raised in several reports [45,58,59,60]. Since another ophiovirus has been reported to be transmitted by the soil fungus *O. brassicae* [61], the possibility of such mode of transmission for CPsV was investigated as a first step. The results effectively showed that an *Olpidium*-like fungus is associated with citrus roots and that CPsV is detectable in zoospores from infected roots (the study was carried out on grapefruit). However, some tests of the addition of viruliferous zoospores to healthy citrus seedlings failed to transmit the virus. Further work is still required to confirm the ability of this fungus to transmit CPsV to healthy citrus [59]. More studies have been conducted to identify the potential natural vector of CPsV. Field trials suggest the implication of some aphid species in CPsV transmission [62,63]. However, a recent study conducted in Uruguay with the brown citrus aphid, *Toxoptera citricida* Kirkaldy (Aphididae), proved the inability of this insect to transmit CPsV, at least under controlled conditions [5]. As CPsV is biologically and molecularly different from the other ophioviruses [15,28], further investigations are required to support or definitively reject the potential implication of other means on the natural spread of this virus. A recent survey, conducted in the main citrus-growing areas in Uruguay to monitor the spread of several viruses and viroids associated with citrus, showed high incidence levels (more than 40%) of CPsV in the prospected fields. This statement corroborates the idea that citrus psorosis remains a graft-transmissible disease that represents a major concern for citrus growers in the country due to its particular and poorly understood dissemination means [5].

## 5. Signaling Pathways in Citrus Psorosis Pathogenesis

Phloem-limited pathogens, including viruses, represent a significant research challenge due to the difficulty in detecting them, whilst infected host plants exhibit variable symptoms after a sometimes long incubation phase [64]. Upon infection, the plant, for its part, initiates a set of basal immune responses through its immune system that recognizes microbial- or damage-associated molecular patterns (MAMPs or DAMPs) [65]. Furthermore, plants have developed specific mechanisms to withstand pathogens, including performed barriers and the induction of elaborate signaling pathways, ensuring the protection against a broad spectrum of pathogens [66]. Such protection is already provided through both signaling and defense molecules, including hormones, RNA, and proteins, which are transported via the phloem [67]. Thus, virus multiplication and propagation within an infected plant is usually restricted by the activation of the small interfering RNA (siRNA) antiviral machinery and/or phytohormone signaling pathway [68].

RNA silencing mechanisms have been suggested to be involved in the citrus response to CPsV. It has been shown, through transcriptomic analysis, that CPsV infection promotes a drastic downregulation of *Citrus sinensis* L. endogenous miRNAs followed by an upregulation of its target genes, the transcription factors *Squamosa promoter-binding protein-like* (SLP) and *Scarecrow-like 6* (SCL6). The latter was involved in the activation of programmed cell death and the reduction of chlorophyll biosynthesis. Therefore, the vast amount of both transcripts may contribute to the onset of necrosis and chlorosis symptoms on CPsV-infected plants [16,69]. Additionally, it has been proven that the CPsV 24K protein physically interacts with citrus miRNA precursors, thus affecting their processing and subsequent miRNA accumulation and target expression. The downregulation of miRNAs is associated with the development of symptoms in CPsV-infected plants of sweet orange [16]. Subsequently, ten putative targets from four downregulated miRNAs of the same host have been predicted. Six of them have been validated through the analysis of leaf degradome data. Their expression in infected samples was higher compared to healthy tissues and was positively correlated with symptom severity [69]. Therefore, the CPsV 24K protein is considered as a potential viral suppressor of RNA silencing (VSR) and effector of CPsV. This is mainly due to its ability to affect the miRNA pathway, whose components are shared by antiviral silencing, and to induce the expression of genes probably involved in the development of disease symptoms [70].

The activation of complex phytohormone signaling networks is a common defense response used by plants to surmount pathogenic threats [71]. Unsurprisingly, pathogens have evolved in particular ways to manipulate or subvert the signaling pathways for their benefit [72], thus inducing pathogenesis. Although plant viruses contain relatively simple genomes, the molecular basis of the mechanisms by which they infect their hosts and the signaling components involved in host resistance are not well-defined [73], and CPsV is no exception. Furthermore, the mechanisms underlying molecular and biochemical changes during compatible and incompatible plant–virus interactions are only beginning to be deciphered, including changes in proteomic profiles induced by virus infections [74]. It is possible that signaling pathways may occur upon the infection of citrus by CPsV. However, to our knowledge, there are no studies describing the phytohormone signaling networks in CPsV-infected plants and their potential involvement in plant defense. Therefore, additional studies are needed to shed light on the CPsV–citrus interactions which can regulate phytohormone pathways, thus leading to disease onset and hormone-mediated signaling and responses to the virus. A deeper understanding of plant anti-CPsV immunity may facilitate the development of innovative and promising approaches to control psorosis disease.

## 6. Methods to Detect the Disease

### 6.1. Biological Indexing and Cross Protection

Biological indexing requires one year and is currently the only and validated technique applied for citrus psorosis diagnosis [75]. In some cases, psorosis is completely symptomless in field trees over several years, and biological indexing is, therefore, an absolute requirement to verify that citrus trees are not infected with CPsV. Indexing is also an extremely important technology for selecting source trees for commercial propagation since it can detect other graft-transmissible pathogens [2]. CPsV diagnosis by indexing has been reported on several indicator plants, including Madam Vinous sweet orange, Eureka lemon, citron grafted on a rough lemon rootstock [48], Pneapple sweet orange, and *Citrus excelsa* Wester seedlings [46]. The glasshouse temperatures for psorosis indexing should be maintained within a relatively cool temperature range at 16–18 °C minimum and 27–30 °C maximum. If the requirements set out for indexing are met, and plants are vigorous and in good growing condition, the indexing will be successful and specific symptoms will appear. The first symptom induced by the disease on the favorite sweet orange indicator varieties of Madam Vinous or Pineapple is the wilting and dieback of the new emerging shoots. This symptom is known as “shock reaction” [2], and it starts to appear during a very brief time interval (around 15 days) [16]. The young leaves will dry up and drop off [2]. In addition to shock reaction, oak-leaf pattern symptoms have also been reported on different woody indicator plants following their infection by distinct CPsV isolates [35,57]. It has been shown that symptoms expression on indicator plants varies according to CPsV isolates and temperature conditions [48,57].

CPsV is associated with the development of “shock reaction” in many indicator plants and leaf mottle and yellows in citron under cool conditions. There is a considerable variation in the leaf mottle produced by different CPsV isolates (from a mild general mottle in the young flush to an intense chlorotic spotting and variegation in mature leaves) [48]. Regarding the effect of temperature on symptom expression, it has been found that cool temperature promotes the onset of “shock reaction” in young shoots and intensifies chlorotic flecks and spots in young leaves, whereas warm temperature tends to inhibit shock and cover-up leaf symptoms. It has also been proven that temperature regulates the viral load, thus affecting symptom expression; temperature increase raises the RNA silencing response of citrus plants and reduces viral accumulation [76]. It is important to emphasize that the single use of biological indexing in the diagnosis of psorosis disease is not enough since some leaf symptoms, like chlorotic leaf flecking and spotting in the following flushes, are also induced by other graft-transmissible pathogens [32] such as citrus leaf rugose virus (Bromoviridae; *Ilarvirus*; CLRV) and citrus tatter leaf virus (Betaflexiviridae; *Capillovirus*; CLRV) [3]. Therefore, a specific CPsV diagnosis also requires a cross-protection test using PsB [32].

Cross-protection is known as an acquired immunity phenomenon by which a mild virus isolate can protect a plant against economic damage induced by a severe challenge isolate of the same virus [77]. In the case of psorosis disease, PsA and PsB are considered as the mild virus and severe challenge isolate, respectively [32,37]. Therefore, plants containing the PsA isolate will protect against a challenge with lesion PsB inoculum [2]. The cross-protection test has been subsequently applied as a supplement to biological indexing in psorosis diagnosis in several studies [46,47]. Since biological indexing is a slow and costly procedure that requires special skills and facilities, the recourse to the development of faster diagnosis methods, as an adjunct to currently available indicator plant indexing methods, is imperative [44,78].

Although biological indexing is considered as the only validated technique for the diagnosis of citrus viruses including CPsV [75], its results remain incomplete. This is based on the fact that its sensitivity to detect CPsV varies depending on the viral isolates associated with the disease. Thus, the probability of occurrence of false negatives, with this technique, is high, especially when using one woody indicator plant and when the requirements set out for indexing are not fulfilled [48,57]. This technique should, therefore, be complemented by at least one other diagnostic method to confirm its findings.

### 6.2. Antibodies–Antigen-Based Methods

Enzyme-linked immunosorbent assay (ELISA) and direct tissue immunoblot assay (DTBIA), the most common detection methods for plant viruses, were shown to be able to identify CPsV sensitively and reliably [33,79,80]. This is basically due to the successful trials implemented to produce polyclonal and monoclonal antibodies to CPsV which were highly effective in detecting several CPsV isolates from many sources and originating in many countries [31,44,81,82]. Since the successes of those trials, these serological methods have had high success for routine and large-scale diagnosis of CPsV [83]. Additionally, the developed antibodies have been used to detect CPsV virions using immunosorbent electron microscopy (ISEM). The results indicate that CPsV can be readily detected by this technique [49].

Comparing these serological methods to each other revealed that the triple antibody sandwich (TAS-) ELISA is at least five times more sensitive than the double antibody sandwich (DAS-)ELISA in detecting CPsV isolates (Table 1). This test allowed CPsV detection even when the viral concentration is low, the year season is unfavorable (in the wintertime), or composite samples including some healthy material are used. Under controlled conditions, CPsV was still detectable at a leaf extract dilution of 1/6,250 in DAS- and at 1/31,250 in TAS-ELISA [82]. It has also been shown that CPsV detection by DTBIA was less consistent than TAS-ELISA, especially in old leaves. TAS-ELISA can be used in any season as pointed out earlier, whereas DTBIA requires young tissue [46]. ISEM results were similar to those achieved by different ELISA formats using antibodies or even other procedures detecting viral RNA such as reverse transcription polymerase chain reaction (RT-PCR) and molecular hybridization [49]. Considering the high variability of the CP gene of CPsV [27], and to take into account all CPsV isolates while enhancing the inclusiveness of serological tests, it is recommended to include a mixture of MABs when applying this testing procedure [46]. Additionally, it has been shown that the use of an alternative enzyme to alkaline phosphatase (AP), the horseradish peroxidase (HRP), in TAS-ELISA improves the test analytical sensitivity and reliability with reduced time. Moreover, TAS-ELISA-HRP sensitivity was comparable to that of RT-PCR [33]. The high specificity of the TAS-ELISA was reported in several studies carried out in Argentina, Spain, and Italy. Optical density (OD) values obtained for non-infected samples were always low and similar to those obtained with phosphate-buffered saline (PBS buffer) [33].

### 6.3. Nucleic Acid-Based Methods

A comparison of the RT-PCR and DAS-ELISA results suggests that both methods can be applied to detect a wide range of CPsV isolates, but that viral variation in the field might cause problems for any single diagnostic test [42,44]. To address issues of the major limitations of some serological tests (low titer, sampling season, uneven distribution of the virus in infected plants, etc.), molecular assays based on RT-PCR and hybridization have been developed for the detection of CPsV. These RNA-based detection methods have been set up notably through the availability of the complete genomic sequence of two CPsV isolates and partial sequences of many others [12]. All RT-PCR and related tests developed to detect CPsV are presented in Table 2. To address the problem of non-detection of some CPsV heterologous isolates by the RT-PCR previously developed by Garcia et al. [44], a highly sensitive and reproducible heminested RT-PCR assay was developed. The new test can detect CPsV in a small amount as an equivalent of 10^10^ of the original sample. This represents a considerable improvement (of 10^6^-fold) compared to the previously reported sensitivity [78]. An improved RT-PCR test was developed by Achachi et al. [84] based on the assay previously reported by Martín et al. [49]. It has been shown that this test is more sensitive and more reliable than DAS-ELISA in detecting CPsV in field samples. Additionally, a difference in the sensitivity was reported by comparing three primer pairs, CPV1/CPV2, Ps65/Ps66, and CPsV-f/CPsV-r (Table 2). The primer pair Ps66/Ps65, which targets the most conserved region of the CP gene, enables the detection of the virus in 22 out of 30 samples tested, whereas CPV1/CPV2 and CPsV-f/CPsV-r could detect CPsV only in 11 and 12 samples out of 22 samples, respectively [84]. Since several RNA and DNA viruses can infect a single host, a multiplex RT-PCR assay was developed to allow the simultaneous detection of seven viruses associated with citrus, including CPsV. This test is considered as a useful rapid method that will aid in the production of virus-tested citrus plants for certification purposes [85]. Real-time RT-PCR is a well-established diagnostic method applied to many pathogens and crops, including woody plants such as citrus and its relatives [75]. Recently, many studies have shown the reliability of this tool even for the simultaneous detection of several viruses in citrus [75,86]. It has been shown that the real-time RT-PCR assay is 100 times more sensitive than conventional RT-PCR when tenfold serial dilutions were prepared using total RNAs from CPsV-infected plants [34]. Additionally, rapid and accurate multiple detections of CPsV and two other citrus viruses were reached applying a triplex one-step real-time RT-PCR. Therefore, it has been suggested that this test may be helpful as a tool to support quarantine, eradication, and certification programs. This is mainly because this test is faster, more sensitive, relatively easier to perform, and more cost-effective than the conventional detection procedures [34]. Other triplex real-time RT-PCR assays have been developed by Osman et al. [86] to detect CPsV simultaneously with two other citrus-infecting viruses. The developed tests have been proven to ensure the detection of each of the three targeted viruses both in single infected samples and in a mixture of infected samples. Each of the triplex real-time RT-PCR assays showed the same level of robustness than that of the singleplex ones. Triplex real time RT-PCR is, therefore, considered as a good tool streamlining the detection of multiple viruses at citrus nurseries by reducing time and labor without affecting both sensitivity and specificity [86]. A SYBR Green real-time RT-PCR protocol was developed by De Francesco et al. [75] and compared to ELISA, the most sensitive and universal method used to date in CPsV diagnosis [46]. It has been proven that the real-time RT-PCR sensitivity was three orders of magnitude higher than that of TAS-ELISA [75]. The specificity of the tests described above was confirmed by including healthy controls and/or plant tissue infected with other citrus viruses and viroid. No amplification was obtained with these controls [34,75,78,84,86]. Table 1 provides a comparison of the methods described above in terms of both sensitivity and specificity.

Dot-blot hybridization protocols have been developed to detect CPsV [25,49,87]. Although its results that could be 100% or less coincident with those of other tests like RT-PCR, DTBIA, DAS-ELISA, and TAS-ELISA [25,49], dot-blot hybridization is considered as an outdated technique to detect CPsV.

The current advancements in molecular biology allow the direct detection, identification, and discovery of known and novel viruses in multiple plants without antibodies or prior knowledge of viral sequences [88]. Over the last decade, an unprecedented number of viruses have been discovered using high-throughput sequencing (HTS), which led to the advancement of our knowledge on viral diversity in nature, particularly apprehending and deciphering the virome of many crops [89]. This approach has been often applied to study and identify new viruses in fruit tree species, including citrus [90,91,92]. The electronic-probe (e-probe) bioinformatics approach has been developed and evaluated recently for its ability to detect several citrus viruses simultaneously in HTS data. The research included 11 citrus-infecting viruses of global economic importance. This approach has been useful for the rapid and simultaneous identification of 11 recognized viruses, including CPsV. This latter has been detected in only one field sample and the result was confirmed with de novo assembly-based similarity searches and RT-PCR [93].

The availability of advanced and sensitive diagnostic methods for detecting the CPsV reduces the need to use biological indexing, which requires more time to detect the same virus. 

**Table 1:** Summary table of the main findings from the different CPsV detection tests.

Tested Methods Main Findings	References
**DAS- and TAS-ELISA**	[82]
TAS-ELISA is five times more sensitive than DAS-ELISA. Threshold of sensitivity: CPsV is still detectable in a leaf extract dilution 1/31,250 for TAS-ELISA and only 1/6,250 for DAS-ELISA, with optical density (OD) of 0.036 and 0.027, respectively. High specificity of TAS-ELISA in non-infected samples. It always gives low OD values (0.006).	
**RT- and Heminested RT-PCR** Heminested RT-PCR allows the detection of eight CPsV isolates, whereas no signal was detected with the conventional RT-PCR using only two primers. Threshold of sensitivity: CPsV is still detectable until a dilution of 10^−5^ with heminested RT-PCR.	[78]
**DAS-, TAS-ELISA, and DTBIA** DTBIA for CPsV detection in the same trees is reliable only when using young shoots. It is less consistent in old leaves. TAS-ELISA sensitivity is two to eight times higher in young shoots compared to old ones. High specificity of ELISA tests: the ELISA readings of positive controls were within the range 0.300–0.550, and those of the negative controls were within the range 0.002–0.013.	[46]
**TAS-ELISA-HRP and TAS-ELISA-AP** TAS-ELISA-HRP readings are at least two times higher than those of TAS-ELISA-AP after two hours of incubation. TAS-ELISA-HRP is highly sensitive and comparable to the level of the RT-PCR. It gives the same results as RT-PCR using primers CPV1 and CPV2 (Table 2). High specificity: TAS-ELISA in non-infected samples always gives low OD values.	[33]
**One-Step RT-PCR and DAS-ELISA** One-step RT-PCR is more sensitive than DAS-ELISA. (One-step RT-PCR allows the detection of 22/30 positive samples, whereas DAS-ELISA allows the detection of only 7/30 positive samples and gives 15/30 false negatives.) The primer pair Ps66/Ps65 is more sensitive than two other primer pairs (Table 2). Ps66/Ps65 allows the detection of 22/30 positive samples, whereas CPV1/CPV2 and CPsV-f/CPsV-r allow the detection of only 11/22 and 12/22, respectively.	[84]
**Real-Time RT-PCR and RT-PCR** Real-time RT-PCR is 100 times more sensitive than conventional RT-PCR. High specificity: no amplification was obtained with healthy controls or with plant tissue infected with other citrus viruses and viroid.	[34]
**Real-Time RT-PCR and TAS-ELISA** Real time RT-PCR sensitivity is three orders of magnitude higher than that of TAS-ELISA. TAS-ELISA gives eight false-negative field samples. Two of these eight samples showed symptoms of psorosis; the remaining samples were asymptomatic. Threshold of sensitivity: positive TAS-ELISA readings were only obtained with undiluted samples or samples diluted by 10^−1^. In contrast, real-time RT-PCR was positive up to the 10^−4^ dilution.	[75]
**Singleplex and Triplex Real-Time RT-PCR**	[86]
No significant differences in detection between singleplex and triplex assays in CPsV detection. The specificity was not affected by the inclusion of the three singleplex assays in a multiplex real-time RT-PCR reaction. No amplification was detected with samples from healthy citrus or water control.	

**Table 2:** Primer sequences and their annealing temperature (T_m_), primer/probe location, and expected size of PCR products for each primer pair when used to amplify CPsV by RT-PCR and related tests.

Name of RT-PCR Test Primer/Probe Name	Sequence	T_m_ (°C)	Targeted RNA/Region (Genomic Coordinates)	Size of the Expected Product	References
**RT-PCR**					
Primer 1	5′-ACAATAAGCAAGACAAC-3′	45	RNA1 (DN ^a^)	218 bp	[44]
DN	5′-CCATGTCACTTCTATTC-3′				
CPV1	5′-GCTTCCTGGAAAAGCTGATG-3′	50	RNA3/CP (665–684 ^a^)	600 bp	[25]
CPV2	5′-TCTGTTTTTGTCAACACACTCC-3′		RNA3/CP (1243–1264 ^a^)		
Ps65	5′-TGCCATCTGGAGTGAGGCT-3′	45	RNA3/CP (1182–1200 ^b^)	430 bp	[49]
Ps66	5′-TCGAAGCTGTATGATGGTGA-3′		RNA3/CP (768–787 ^b^)		
ConsF	5′-ACAAAGAAATTCCCTGCAAGGG-3′	58 and 59–60 for singleplex and multiplex test, respectively.	RNA3/ CP (766–1200 ^c^)	411 bp	[85]
ConsR	5′-AAGTTTCTATCATTCTGAAACCC-3′				
CPsV-f	5′-TGAGGAA/GTTGAGCCATGC-3′	58	RNA3/CP ^d^	390 bp	[42]
CPsV-r	5′-CCATCTGGAGTGAGGCTGTA-3′				
**Heminested RT-PCR**					
Primer 1		46 and 47 for the first PCR done with primers 1–7 and the heminested PCR done with primers 6–7, respectively.	RNA1 (DN ^a^)	195 bp	[78]
Primer 6	5′-GAGGAAGGTATTTCCATAGG-3′				
Primer 7	5′-CCTATTAATGATAATTGCAC-3′				
**Real-time RT-PCR**					
CPV200f	5′-GCWGGWAATCGRTCTGTGAGRTAT-3′	58 and 60 for singleplex and multiplex tests, respectively.	RNA3/CP (200–223 ^e^)	106 bp	[34]
CPV287r	5′-AGCAAWGGCATCARGGAYTC-3′		RNA3/CP (287–306 ^e^)		
CPVp	5′-Cy5b-TCYCCTGCTGTTGGWGCAACTYC-BHQ1-3′		RNA3/CP (263–285 ^e^)		
CP1cf	5′-GTTCAAGATGGAGCAAGTTGATGG-3′	56	RNA3/CP (738–850 ^f^)	113 bp	[75]
CP3r	5′-GAGACCCTTGTGTAAAAACCAGCAC-3′				
CPsV-792 F1	5′-TCACAAATCAGTGAGGAATTGAGC-3′	60 for singleplex tests. For multiplex tests, the T_m_ was defined according to the manufacturer’s recommendation.	RNA3/CP (792–816 ^g^)	154 bp	[86]
CPsV-791 F2	5′-CACAAATCAGTGATGAATTGAGCC-3′		RNA3/CP (793–817 ^g^)		
CPsV-946 R1	5′-GCAAACCCAGCATATCTCACAG-3′		RNA3/CP (947–925 ^g^)		
CPsV-946 R2	5′-CGCAAACCCAGCATATCTTACAG-3′		RNA3/CP (948–925 ^g^)		
CPsV-851 p-VIC	5′-TCTCAAGATTGATATAGACAAC-3′		RNA3/CP (851–873 ^g^)		

DN: data not shown. ^a^ Florida isolate CPsV-4; EMBL accession number AF060855. ^b^ Based on regions of the CP gene highly conserved in 23 psorosis isolates from different geographic areas. ^c^ Florida isolate CPsV-6. ^d^ Based on the most conserved region of the CP of more than 20 sequences found in public databases. ^e^ Based on an Italian isolate CRSA-UBAps101; EMBL accession number AM409317. ^f^ Based on an Argentinian isolate CPsV 90-1-1; EMBL accession number FJ495195 (viral complementary RNA). ^g^ Based on a partial sequence of the CP; EMBL accession number AF036926.

## 7. Strategies to Control the Disease

Like many plant virus diseases, the first essential requirement for controlling psorosis disease is prevention. The meticulous selection of healthy propagating material is, therefore, the key issue to stop the spread of citrus psorosis [1]. The control of psorosis in any citrus-growing area requires the establishment of three related but separate programs: sanitation, quarantine, and budwood certification and indexing. Sanitation programs aim to recover healthy plants from selected local cultivars. Regarding quarantine programs, the objective is to guarantee that the foreign imported varieties are free from regulated pests and diseases. Finally, the main aim of certification programs is to ensure that the sanitary status of the initial material is conserved during commercial propagation stages of process at the nurseries [94]. Psorosis was the first citrus disease for which both eradication and budwood certification programs were launched to prevent its economic damage [12].

### 7.1. CPsV Sanitation

Among sanitation methods, somatic embryogenesis, from stigma and style cultures, has been tested for its ability to ensure the elimination of CPsV and the production of healthy citrus stocks. This method was applied to eliminate CPsV from some citrus species including common mandarin, sweet orange, and Dweet tangor. The results showed that the virus was detected by DAS-ELISA in both explants and embryogenic callus, but was not detected in any of the plants obtained from somatic embryos, two years after their regeneration [95]. The same results have been reported by El-Sawy et al. [96] who proved that the somatic embryogenesis via stigma was a good tool for the production of CPsV-free citrus plants. Thermotherapy is another method that can be applied to eliminate the virus from propagative budwood. It can be carried out in a chamber at 40 °C for 16 h with lights and 30 °C for 8 h in the dark for a period of 8 to 12 weeks [2]. Sanitation by in vitro shoot-tip grafting has also proved to be a very effective method for citrus graft-transmissible disease elimination including psorosis (success rate of about 60%) [64,97]. However, this technique should be combined with thermotherapy to get rid of CPsV [64]. Whatever chosen sanitation method, biological indexing must be conducted to assure that propagative material (i.e., budwood) is CPsV-free [2].

### 7.2. Plant Biotechnology and Genetic Engineering for Resistance

Since no sources ensuring natural resistance to CPsV have been found, and pathogen-derived resistance (PDR) is considered as an efficient alternative to control viral infection in some plant–virus systems, the recourse to transgenesis to manage CPsV was a necessity and a promising alternative to exploit. Trials carried out on sweet orange lines genetically transformed with the CP gene of CPsV and challenged with a CPsV isolate have so far failed. Neither resistance nor tolerance has been expressed in transgenic lines after one year of observations. Conversely, typical psorosis symptoms have been observed in all of them after inoculation [98]. A year later, it was reported, by using a herbaceous model plant, that post-transcriptional gene silencing (PTGS) was the mechanism conferring resistance to CPsV. PTGS is a mechanism of plant defense against viruses that proved to be induced by transgenic expression of virus-derived sequences encoding hairpin RNAs. In the case of resistance to CPsV, PTGS induction occurred efficiently using the CP-derived sequences [99]. A few years later, it was proved that the generation of transgenic sweet orange lines expressing, this time, hairpin RNA transcripts corresponding to the CP gene (ihpCP-transcripts) provided a high level of CPsV resistance. This study is considered as the first report of resistance in citrus plants against a -ssRNA virus as the CPsV. The key factors associated with this resistance are the pre-activation of the RNA-silencing processes and the accumulation of a minimum of siRNA molecules targeting the viral genome [100]. Furthermore, a virus-induced gene silencing mechanism (VIGS) has already been pointed out by the presence of specific siRNA for RNA1, 2, and 3 of CPsV in infected plants [76]. Further investigations revealed that the strong resistance against CPsV previously reported by Reyes et al. [100] is durable and stable since the transgenic line remained resistant two years later and even after re-inoculation [101]. Furthermore, recent scientific works indicate that transgenic line expressing ihpCP-transcripts in the interstock confers tolerance to CPsV in the non-transgenic scion. It is established, using a triple plant system generated by grafting, that ihpCP-transcripts translocate through the graft, conferring silencing induction in the scion by the expression of ihpCP in the interstock and the protection to the non-transgenic scion against CPsV. These results corroborate the idea that grafting is a promising biotechnological alternative to protect woody plants against viral infection in vegetatively propagated material [102].

### 7.3. Breeding for Resistance

Since the use of transgenic plants is restricted in many countries, searching for tolerant and resistant citrus cultivars obtained by breeding programs remains a necessity. In this context, potential sources conferring natural resistance against CPsV were investigated by graft inoculating different cultivars and hybrids of *Citrus* and related genera with the virus and then monitoring symptom expression, CPsV infection, and viral loads. Graft inoculation has been done using CPsV-infected sweet orange rootstock (as susceptible infected host) to ensure a continuous virus supply to the tested genotypes. The results have established that some genotypes (*Microcitrus inodora* F.M. Bailey and *Fortunella hindsii* Swingle) were tolerant to the virus and showed symptomless infections, while others (Cleopatra mandarin, *Poncirus trifoliata* (L.) Raf. and Carrizo citrange seedlings) displayed partial resistance induced by a hypersensitive-like reaction that prevents plant infection or delays viral spread and accumulation. The observed resistance in these genotypes is CPsV isolate-independent [103].

## 8. Disease Situation in the Mediterranean Region: Focus on Morocco

Citrus psorosis is a widespread disease in the Mediterranean region. CPsV has been reported in most Mediterranean countries and is one of the most prevalent citrus viruses in the region [8]. The development of reliable diagnostic methods facilitated extensive surveys for CPsV in different parts of the region. The virus was successively identified in many countries including Morocco [83], Palestine [104], Italy [27], Tunisia [105,106], Egypt [57], Turkey [35,107], Greece [108], Albania [109], Cyprus [110], Malta [111], Algeria [112], Spain [28], Israel [113], Lebanon, Jordan, and Syria [52]. The use of budwood taken from infected trees is assumed to be the main explanatory factor behind the widespread of this disease in the Mediterranean area [52]. Whenever surveys were conducted, CPsV appeared to be one of the most common citrus viruses. In Tunisia, the CPsV prevalence was determined from 575 citrus samples randomly collected in 2014. Some citrus trees from which leaf samples were taken presented a severe or mild symptom of bark scaling. The virus prevalence was high, and ranged between 20 and 47%, according to geographical location, citrus species, and varieties [106]. In Algeria, in an extensive survey carried out from 2004 to 2006, CPsV was detected in 60% out of a total of 467 citrus samples [112].

**Figure 3:**
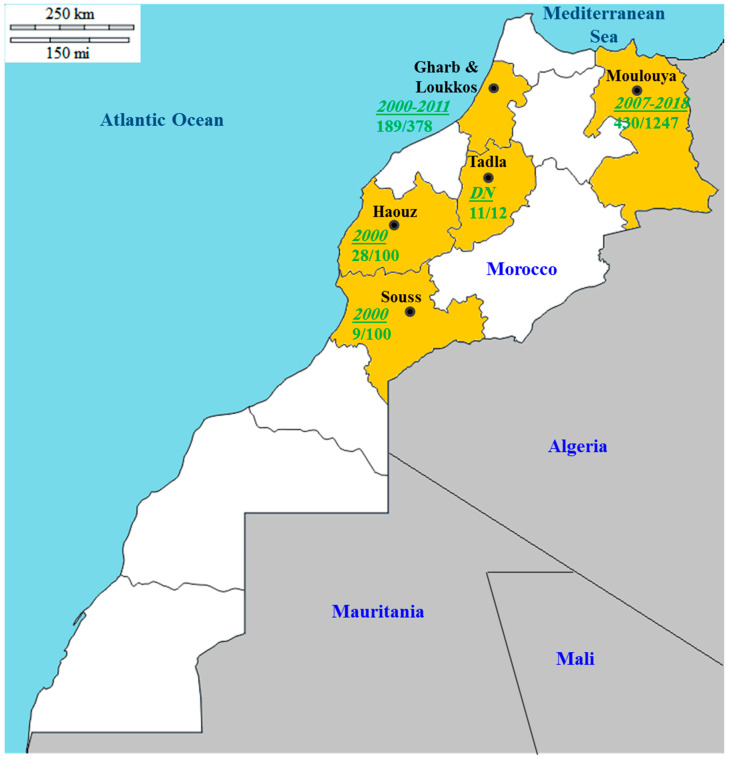
Map of Morocco showing the five regions (highlighted in orange) in which field surveys have been carried out in the main citrus-growing areas to monitor CPsV spread. The green numbers correspond to the years of sampling (italic and underlined) and the number of positive samples to the total collected per area. DN: data not shown.

In Morocco, psorosis constitutes one of the major viral diseases of citrus [114]. This disease is prevalent in old orchards and can be found in all citrus species and varieties [115,116]. Furthermore, it has been proved impossible to find old orange trees (more than 20 years), with virus-like symptoms, lacking the virus associated with this disease [116]. Widespread CPsV has been partly explained by the grafting-on practice extensively applied by Moroccan growers [117]. The main citrus-growing areas of Morocco investigated from 2000 to 2018 for CPsV monitoring are summarized in Table 3 and illustrated in Figure 3. It is clear that CPsV is omnipresent in almost all citrus-growing areas of the country with relatively high prevalence (9–92%). It is clear from Table 3 that Gharb and Moulouya are the main areas where extensive samplings have been carried out and where CPsV has been detected frequently, with an average prevalence of 50%. The virus was also detected in the other three citrus-growing regions. Furthermore, the virus can infect different citrus species and varieties. Concerning CPsV genetic analysis, a first sequence comparison among 34 Moroccan isolates collected in the three main citrus-growing areas of Morocco (Gharb, Tadla, and Moulouya) showed limited variation in the CP gene [30]. Further comparisons of these isolates with samples from Spain, Italy, Argentina, California, and Florida disclosed four groups: group I includes isolates from the three citrus-growing areas of Morocco; group II includes isolates from Gharb, Moulouya, Spain, and Italy; group III includes isolates from Tadla and Moulouya; while group IV includes the Argentinean, Floridian, Californian, and Italian isolates. The genetic diversity within groups is low and not structured by the geographic origin of each isolate. It is important to emphasize that this genetic structure suggests a displacement of some CPsV isolates among geographically isolated subpopulations. This assumption is made since an identical genetic structure was observed among isolates from various areas which are separated by several hundreds of kilometers. The intense exchange of citrus propagation material, including CPsV-infected material, between distant geographic regions of Morocco seems to be behind this observation. Furthermore, the occurrence of high gene flow between the Moulouya and Tadla regions has been highlighted. This gene flow could have contributed to shaping the genetic structure of the population, and could also be responsible for the low genetic variability observed within the Moroccan isolates [30].

## 9. Conclusions

The presumed widespread of citrus psorosis in most citrus-growing areas was mainly based on the unique observation of CPsV symptoms which is reported not sufficient to make such a conclusion [49]. Indeed, a still undefined small proportion of trees expressing “psorosis-like” bark scaling symptoms shows no evidence of the presence of the virus [4]. In other words, the idea of a non-CPsV psorosis-like disease of unknown etiology is emerging [4]. HTS technologies can be applied to decipher this issue and shed light on the disease etiology in these cases. Additionally, stringent testing for CPsV detection has generally been lacking [4]. Therefore, CPsV incidence might be more limited than it initially appears to be and in some cases, the reported symptoms might be linked to causes other than CPsV. It should be recognized, however, that CPsV incidence might be raised since some trees without bark scaling were shown to be CPsV-infected [49].

It is not easy to select one diagnosis method over another even when their results are consistent. The rationale of choice will depend on the diagnosis purposes and the facilities available at each laboratory. DTBIA and molecular hybridization may be advantageous for large scale epidemiological studies as they do not require a specific sample processing. Furthermore, tissue prints can be conducted in the field for both methods, thus avoiding the transport of infected plant material between regions. Membranes can also be stored for a long period of time before processing [49]. Although ELISA is simpler to handle, it is much less sensitive than RT-PCR. However, to certify the health of source plants, RT-PCR is considered as a more appropriate method [79]. Briefly, the advantages and disadvantages of different diagnostic methods should be discussed in advance without forgetting that the use of more than one method is always the safest decision in most diagnosis situations [49]. Despite the availability of serological and molecular tools for CPsV detection, biological indexing of donor plants on sweet orange seedlings will continue to be used in certification programs since it is still the unique procedure allowing the detection of other graft-transmissible citrus pathogens causing young leaf symptoms [46,49]. Given its capacity to both detect known viruses and discover new viral species or distant isolates of known viruses, HTS could potentially be applied to control the movement of plant material between countries [120]. This is because HTS of either the transcriptome (RNA-Seq) or sRNAs has been considered as a promising and powerful approach in detecting both RNA and DNA viruses [121,122]. This approach can, therefore, be adopted to better understand psorosis disease and to explore CPsV-citrus interactions. This approach will also help to identify putative viruses associated with citrus psorosis-like symptoms with unknown etiology and compare the frequency and diversity of viruses between CPsV-symptomatic and -asymptomatic plants grown in a CPsV-infected region, as it has been already carried out for citrus sudden death disease [91].

Control of the sanitary status of donor plants for the production of CPsV-free propagating material is crucial. The deployment of shoot-tip grafting in vitro, in combination with thermotherapy or somatic embryogenesis from stigma and style cultures, has proved effective for eliminating CPsV from plant propagating material [4]. The fact that most (if not all) commercial varieties of sweet orange, mandarin, and grapefruit are sensitive to CPsV infection is a key constraint for the use of natural resistance [12]. Although partial resistance has been recently reported, its incorporation in commercial citrus varieties by conventional breeding programs seems to be a much more complex task. This is closely linked to the fact that this resistance has been detected only in trifoliate orange, Carrizo citrange, and in Cleopatra mandarin [103]. An alternative approach for obtaining CPsV-resistant varieties would be the production of transgenic plants expressing CPsV genes leading to the activation of RNA-silencing processes upon CPsV infection [12]. In other words, the development of transgenic citrus lines carrying parts of the CPsV genome may constitute a promising biotechnological approach aimed at eradicating psorosis [4]. Additionally, highly resistant transgenic sweet orange plants expressing ihpCP-transcripts have been developed [100]. The stability and durability of the resistance conferred by this construct have been confirmed [101]. Furthermore, it has been demonstrated that ihpCP-transcripts translocate through the graft from interstock to scion, activating the silencing of the CP mRNA target. This means that resistance could be transmitted bi-directionally in a triple plant system generated by grafting. This approach is therefore considered as a promising biotechnological alternative to protect woody plants against viral infections in vegetatively propagated plants [102]. Currently, genome editing ensured by clustered regularly interspaced short palindromic repeats (CRISPR)-based systems is considered as a crucial genetic manipulation tool that can be applied to numerous crops, including citrus. It is in fact established that CRISPR systems, such as CRISPR/CRISPR-associated protein (Cas)9 and CRISPR/Cpf1 systems, can provide a promising new corridor for producing citrus cultivars expressing resistant to different pathogens [123].

Regarding the current situation of CPsV in Morocco, this review provides a general overview of its spread in the Moroccan citrus-growing areas. Preventing the introduction and spread of psorosis disease in the Moroccan citrus orchards could be ensured by using virus-free (certified) planting material, disinfecting pruning tools, regularly monitoring of citrus groves for an early detection of the disease, and grubbing up of diseased trees. This review pointers for new research avenues in psorosis disease either in Morocco or elsewhere. These research areas could include for instance the characterization of CPsV isolates, searching for potential vectors and secondary hosts, and developing sustainable control strategies. Studying functional genomics through transcriptomic analysis and/or proteomic approaches in citrus–CPsV interaction would be an interesting approach to shed more light on the full mechanisms underlying the complex and varied events associated with such interactome, and thus help to develop novel diagnostic methods and plant protection strategies. This further advanced research will expand our understanding of CPsV epidemiology and the mechanisms behind its spread across the world in general and Morocco in particular, and could potentially help in devising innovative management strategies of the virus.

## Figures and Tables

**Table 3:** Results summary of the known field surveys carried out in the main citrus-growing areas of Morocco to monitor CPsV spread.

Date (Collection Period)	Locality (Number of Visited Groves)	Plant Material	Symptoms	Diagnostic Techniques (Tested Tissue)	Tested Varieties	CPsV * Prevalence (Positive/Total)	Positive Varieties (Percent (%) of Infection)	References
2000 (March)	Gharb (2)	Five flowers, or six mature leaves from trees that were not in bloom.	Bark scaling and psorosis-like symptoms on the leaves (mottling, flecking, and ringspots).	DTBIA (ovary tissue) and ELISA (mature leaves from non-flowering trees).	Maroc-late and Salustiana orange trees.	50% (172/346)	Maroc-late (71%) and Salustiana (36%).	[83]
	Souss (7)				Nour clementine, Ortanique mandarin, Washington Navel, Maroc-late and Salustiana orange, Eureka lemon, and Star ruby grapefruit trees.	9% (9/100)	Ortanique (20%), Washington Navel (20%), Maroc-late (14%) and Salustiana (20%).	
	Haouz (7)					28% (28/100)	Ortanique (50%), Washington Navel (49%), Maroc-late (14%), Salustiana (20%), Eureka (4%), and Star ruby (8%).	
2007 (June)	Moulouya (8)	Young leaves from the four cardinal orientations.	Bark scaling.	ELISA.	Berkane clementine and Washington Navel orange trees.	77% (50/65)	Berkane clementine (22%) and Washington Navel (41%).	[117]
			Vein clearing on leaves.			8% (3/39)		
			Leaves showing oak leaf pattern.			7% (1/15)		
			No symptoms.			6% (5/81)		
2011 (January)	Gharb (DN)	Four young shoots.	Typical psorosis bark scaling.	RT-PCR (leaves) and/or biological indexing.	Maroc-late, Washington sanguine, Washington Navel orange, Sidi Aissa clementine, and sour orange trees.	100% (8/8)	DN	[118]
			Non-scaled trees with young-leaf symptoms.			100% (4/4)		
			Atypical bark scaling.			0% (0/2)		
			Concave gum symptoms.			0% (0/1)		
			Non-scaled trees showing young leaf symptoms.			0% (0/5)		
			No symptoms.			0% (0/4)		
2008–2013 (DN)	Gharb, Haouz, Loukkos, Moulouya, Souss, and Tadla (102)	DN	Randomly and plants showing virus-like symptoms were included in the sampling.	ELISA and RT-PCR.	Navelina, Salustiana, Hamlin, Cadenera, Jaffa (Shamouti), Maroc-late, Vernia, Grosse Sanguine, Sanguinelli, and Tarocco orange trees.	33% (1854/5620)	DN	[119]
DN	Gharb (DN)	Four young shoots.	Bark scaling.	ELISA, RT-PCR (leaves for both techniques), and/or biological indexing.	Maroc-late, Salustiana, and Washington Navel sweet orange and Nules clementine trees.	100% (2/2)	Maroc-late (50%)	[84]
			No symptoms.			0% (0/2)		
			Bark scaling.			100% (2/2)	Salustiana (100%)	
			No symptoms.			100% (1/1)		
			No symptoms.			0% (0/1)	Washington Navel (0%)	
	Moulouya (DN)		Bark scaling.			100% (11/11)	Washington Navel (78%)	
			No symptoms.			43% (3/7)		
			No symptoms.			75% (3/4)	Nules (75%)	
	Tadla (DN)		Bark scaling.			100% (11/11)	DN	
			No symptoms.			0% (0/1)		
2018 (May–July)	Moulouya (37)	Four young shoots.	Bark scaling.	ELISA and RT-PCR.	Berkane, Nour, Muska, Nules, Orograndi clementine and Maroc-late, Navel, and Navelina orange trees.	25% (16/65)	Nour (13%), Berkane (44%), Navel (25%), Navelina (6%), unknown orange cultivar (12%)	[116]
			Virus-like symptoms.			17% (/29/170)	Nour (14%), Berkane (21%), Nules (7%), Navel (10%), Navelina (17%), Maroc late (17%), unknown orange cultivar (14%)	
2018 (July–September)	Moulouya (3)	DN	Bark scaling in the trunk and main branches and internal staining in the underlying wood in citrus cultivars.	ELISA and RT-PCR.	Berkane, Nules, and Orogrande clementine trees.	39% (309/790)	Berkane (DN), Nules (DN) and Orogrande (DN)	[115]

DN: data not shown. * Percentages (%) denote the CPsV prevalence according to the type of symptoms observed per locality.
